# Temporal Dynamics of Soil Virus and Bacterial Populations in Agricultural and Early Plant Successional Soils

**DOI:** 10.3389/fmicb.2020.01494

**Published:** 2020-07-07

**Authors:** Krishnakali Roy, Dhritiman Ghosh, Jennifer M. DeBruyn, Tirthankar Dasgupta, K. Eric Wommack, Xiaolong Liang, Regan E. Wagner, Mark Radosevich

**Affiliations:** ^1^Department of Biosystems Engineering and Soil Science, University of Tennessee, Knoxville, Knoxville, TN, United States; ^2^Department of Statistics, Harvard University, Cambridge, MA, United States; ^3^Department of Plant and Soil Sciences, University of Delaware, Newark, DE, United States

**Keywords:** viral ecology, plant succession, abundance, lysogeny, organic matter, land use, induction

## Abstract

As reported in many aquatic environments, recent studies in terrestrial ecosystems implicate a role for viruses in shaping the structure, function, and evolution of prokaryotic soil communities. However, given the heterogeneity of soil and the physical constraints (i.e., pore-scale hydrology and solid-phase adsorption of phage and host cells) on the mobility of viruses and bacteria, phage-host interactions likely differ from those in aquatic systems. In this study, temporal changes in the population dynamics of viruses and bacteria in soils under different land management practices were examined. The results showed that bacterial abundance was significantly and positively correlated to both virus and inducible prophage abundance. Bacterial and viral abundance were also correlated with soil organic carbon and nitrogen content as well as with C:N ratio. The seasonal variability in viral abundance increased with soil organic carbon content. The prokaryotic community structure was influenced more by land use than by seasonal variation though considerable variation was evident in the early plant successional and grassland sites. The free extracellular viral communities were also separated by land use, and the forest soil viral assemblage exhibiting the most seasonal variability was more distinct from the other sites. Viral assemblages from the agricultural soils exhibited the least seasonal variability. Similar patterns were observed for inducible prophage viral assemblages. Seasonal variability of viral assemblages was greater in mitomycin-C (mitC) induced prophages than in extracellular viruses irrespective of land use and management. Taken together, the data suggest that soil viral production and decay are likely balanced but there was clear evidence that the structure of viral assemblages is influenced by land use and by season.

## Introduction

Microbial communities in Earth’s ecosystems are highly abundant, diverse and active and have critical functions in biogeochemical cycling and ecological processes (Bardgett and Van Der Putten, 2014; Coutinho et al., 2018; Hall et al., 2018; Liang et al., 2019a). Viruses, especially those that infect bacteria and archaea, are even more abundant than their co-occurring hosts and play a significant role in shaping bacterial genetic diversity, community structure, and function (Wigington et al., 2016). Many estimates suggest that up to 50% of the daily autotrophic and heterotrophic bacterial production is countered by viral lysis directly impacting nutrient cycling in the oceans (Wommack and Colwell, 2000). Marine viral abundance is positively correlated with bacterial abundance, and many studies suggest viral infection and lysis influence bacterial abundance and diversity by lysing the most abundant bacterial species, resulting in highly even communities (Thingstad and Lignell, 1997; Riemann and Middelboe, 2002; Weinbauer et al., 2003). Lysogeny has been shown to increase with depth in ocean where host cell abundance (and/or activity) is low, and decrease considerably in more productive coastal systems where host cell abundance (and/or activity) is high (Weinbauer et al., 2003).

In stark contrast to these exciting discoveries in marine ecosystems over the past 3 decades, comparable data defining the roles of viruses in the ecology of prokaryotic communities less available in terrestrial ecosystems despite the importance of soils to global biogeochemical cycles and the overwhelming reliance on soil resources for food, fiber, waste disposal/recycling, and bio-product discovery including pharmaceuticals. However, soil viral ecology research has been gaining increasing interest in light of the high abundance and significant importance of soil viruses in shaping the microbial communities and ecological processes (Emerson et al., 2018; Kuzyakov and Mason-Jones, 2018; Liang et al., 2019b,c).

The first direct counts of agricultural soil viruses using epifluorescence microscopy were reported in 2003 (Williamson et al., 2003). Since that time various aspects of soil viral ecology have been examined in wetlands, forest soils, and extreme cold desert soils of Antarctica (Williamson et al., 2007; Martins et al., 2018; Rodela et al., 2019; Liang et al., 2020). Though viruses appear to be highly abundant in soils, relatively few studies have attempted to determine the extent to which soil microorganisms are infected by viruses. In sheep-grazed pasture soil, the frequency of viral infected cells (FVIC) as determined by transmission electron microscopy averaged 23% significantly higher than estimates for many aquatic environments (Bowatte et al., 2010). A similar investigation of a rice paddy system determined that the FVIC from soil exceeded that of the floodwater ranging from 4.2 to 11.4% (mean of 7.4%). Based on the observed FVIC, these investigators estimated that the fraction of bacterial mortality caused by viral lysis approached 100% (Takahashi et al., 2011). These values, if even close to correct, suggest that soil viruses can exert significant top-down control of their prokaryotic hosts and influence biogeochemical nutrient cycling in terrestrial ecosystems. Indeed, Lee et al. (2012) investigated the phage-mediated microbial loop using stable isotope probing and DGGE analysis of cyanophage g20 type genes in a rice field soil. ^13^C-labeled rice callus was used as a substrate and the involvement of the phage mediated microbial loop was demonstrated *via* detection of ^13^C-labeled g20 DNA by stable isotope probing.

In contrast to marine systems, studies of viruses in natural soils have shown a much wider range in virus to bacteria ratios (VBR) in soils. In addition to being more abundant, terrestrial viral communities appear to be much more diverse than their aquatic counterparts. Land use (agricultural or forested) was found to impact bacterial and viral abundances, as were soil organic matter and water content (Williamson et al., 2005). Among two very different soil types (Antarctic and temperate agricultural soil), inducible fraction (IF) was found to be lower in Antarctic soils and higher in temperate Delaware soils, but no clear correlations were found between lysogeny and soil physical properties (Williamson et al., 2007). While these data, including one other study (Ghosh et al., 2008), suggest that lysogeny is more common among soil bacteria than aquatic systems, the specific soil factors (physical, chemical, season, succession, or cultivation methods) that promote lysogenic interactions among viruses with their hosts are still unknown.

Assessing the overall diversity and responses of viral assemblages to environmental change, whether it be natural (seasonal, successional, or pulse events) or human-induced *via* changes in land use, is challenging since there is no universally conserved marker gene(s) present in viral genomes such as the 16S rRNA gene in prokaryotes. Pulsed-field gel electrophoresis (PFGE) based on size fractionation of double-stranded DNA viruses has been used successfully to generate genetic fingerprints of viral assemblages in aquatic environments where viruses are more readily sampled and concentrated (Klieve and Bauchop, 1988; Wommack et al., 1999; Johannessen et al., 2015). More recently, randomly amplified polymorphic DNA-PCR (RAPD-PCR) has been applied as a means for examining fine-scale changes in the community composition of virioplankton within aquatic environments with greater resolution (Williamson et al., 2008; Winget and Wommack, 2008; Helton and Wommack, 2009; De Corte et al., 2010; Goldsmith et al., 2015). The RAPD-PCR technique has also been used multiple times to successfully characterize soil viral assemblages (Srinivasiah et al., 2013, 2015; Liang et al., 2019b). Metagenomic analysis has revealed that soils may contain a 100-fold greater diversity of viruses than aquatic environments, and there is very little overlap in the genetic composition of soil and aquatic viral assemblages (Fierer et al., 2007; Emerson et al., 2018). While omics-based approaches have provided opportunities for further understanding of viral diversity and ecological roles, challenges remain, such as the public virome database is still under-represented with respect to soil viruses and a large fraction of genes in virome sequences have no known homologs or homologs of unknown function (Brum and Sullivan, 2015; Coutinho et al., 2018).

The objective of the present study was to assess the spatial and temporal variability in viral abundance, community structure and the frequency of lysogeny among soil microbial communities and to correlate these properties to soil edaphic factors such as soil moisture, organic matter content, pH, and land management practices in agricultural and plant successional soils. To that end, the abundance and community structure of viruses and bacteria, VBR, and IF, were determined in agricultural and forest soils at approximately monthly intervals throughout the growing season to obtain a seasonal view of phage-host population dynamics. Determination of viral and bacterial abundance was achieved by using epifluorescent microscopy counting, and the frequency of lysogeny was estimated through mitomycin C (mitC) induction assays. Field monitoring of these parameters was conducted in a series of replicated field plots that are part of the Long-Term Ecological Research (LTER) site at Michigan State University’s Kellogg Biological Station (KBS). The results showed a structural variability of viruses and bacteria communities in different land management sites where seasonal variation in bacterial community was lower than the viral community and induced viral community changed significantly with season. Organic carbon content influenced the viral and bacterial abundances and their IF significantly.

## Materials and Methods

### Site Description and Soil Sampling

Soil samples were collected from the LTER site of W. K. KBS (Michigan State University) at Hickory Corners, Michigan. In 1989, this LTER site was established to study ecological processes in agro-ecosystems, which include a large-scale replicated field experiment with seven main treatments representing different cropping systems as well as several plant successional sites^1^. Prior to the establishment of the LTER site, these lands were uniformly farmed for over 50 years (Robertson, 1997). Soil samples were collected at approximately six monthly intervals from Spring to Fall in 2008. Five main treatments were sampled including corn-soybean-wheat rotations, which were either managed with intensive chemical inputs and conventional tillage (T1), or organically managed with minimal input and winter cover-crops (T4; Table 1). Two early plant successional treatments maintained *via* annual mowing or burning (T7 and T8, respectively) and a mid-successional deciduous forest site (SF2) was also sampled.

**Table 1:** The percentage of soil bacteria infected with mitC-inducible prophage estimated using a calculated or assumed burst size in the five KBS land treatments measured at roughly monthly intervals.

Treatment		T1	T4	T7	T8	SF2
Month	IF					
May	IF^*^	32.28 (16.37)	53.51 (8.95)	66.26 (10.26)	56.53 (4.18)	59.02
	IF_20_^#^	7.3 (6.27)	10.91 (1.16)	1.67 (1.11)	2.54 (1.5)	2.24
June	IF	22.84 (17.72)	64.25 (6.95)	70.19 (6.2)	52.08 (2.57)	50.85
	IF_20_	13.03 (8.76)	4.01 (4.78)	1.48 (1.46)	1.84 (1.03)	2.13
July	IF	42.28 (10.22)	73.68 (7.84)	53.92 (9.07)	28.27 (3.53)	48.98
	IF_20_	1.31 (0.29)	1.12 (0.81)	2.01 (1.30)	2.27 (0.35)	2.1
September	IF	59.22 (5.85)	73.47 (11.95)	77.29 (8.03)	67.18 (9.71)	49.65
	IF_20_	1.8 (0.62)	0.9 (0.24)	2.35 (1.28)	4.43 (1.41)	1.03
October	IF	69.40 (14.70)	42.87 (10.10)	62.39 (4.22)	73.56 (12.52)	56.42
	IF_20_	3.02 (1.88)	2.45 (0.76)	1.38 (0.24)	2.86 (1.69)	3.14
November	IF	65.10 (13.73)	62.85 (7.89)	61.91 (5.45)	45.34 (21.25)	73.75
	IF_20_	1.14 (0.58)	0.90 (0.51)	0.89 (0.29)	0.41 (0.54)	0.62

Values are means of triplicate plots (except for SF2) and numbers in parentheses represent one standard deviation.

* Inducible fraction (IF) estimated as described in Williamson et al. (2007) using a calculated burst size.

# Inducible fraction estimated as described in Williamson et al. (2007) using an assumed burst size of 20 viruses per bacterial cell lysed.

Three soil cores (approximately 2.5 cm diameter, 10 cm depth) were collected at each of the five designated sampling stations on each replicated plot and cores were thoroughly mixed to form one composite soil sample for each plot. Samples were collected in this manner from triplicate plots for each treatment except for the SF2 forest site. The soil cores from each field plot were pooled into Ziploc bags, sieved moist (4 mm mesh) to homogenize the samples, and stored at 4°C until analyzed (typically less than 24 h upon returning to the laboratory). Gravimetric moisture content was determined for each sample immediately upon returning to the laboratory.

### Extraction and Enumeration of Bacteria, Viruses, and Viruses From Induced Prophages

Extraction of bacteria and viruses was conducted upon soil samples’ arrival and procession at the laboratory. Induction assays were performed immediately after the extraction of bacteria. While the extracted bacterial and viral suspensions were archived at −80°C, enumeration of viruses and bacteria was fulfilled within 1 month. Viruses and bacteria were extracted from triplicate subsamples of each treatment plot as described elsewhere (Williamson et al., 2003; Ghosh et al., 2008). Briefly, 5-g samples of field-moist soil were weighed into 25-ml Teflon-coated polyethylene centrifuge tubes, and 15 ml of phosphate buffer containing 1% potassium citrate (containing, per liter, 10 g potassium citrate, 1.44 g Na_2_HPO_4_7H_2_O, 0.24 g KH_2_PO_4_, pH 7) was added. All tubes were horizontally shaken on ice for 30 min and centrifuged at 10,000 × *g* to sediment soil particles. Supernatants were passed through 0.22-μm syringe filters to remove bacteria and small soil particles. Bacteria were extracted separately using Nycodenz density gradient columns as described elsewhere (Ghosh et al., 2008). These bacteria were re-suspended in 5 ml of their respective soil extracts that were prepared by autoclaving 5 g of soil in 10 ml phosphate buffered saline (PBS), and split into two separate aliquots: one for bacterial cell enumeration and the other for prophage induction assays. Virus like particles (VLP) and bacteria were enumerated by epifluorescent microscopy as previously described (Ghosh et al., 2008). For prophage induction assays, aliquots of the Nycodenz®-purified cell suspensions were exposed to mitC at a concentration of 1 μg/ml and incubated in the dark for 16–18 h. After incubation the tubes were centrifuged at 10,000 × *g* for 15 min and the supernatant containing the viruses produced by mitC-inducible lysogenic bacteria were collected by filtering through 0.22 μm polycarbonate filters (Millipore) to remove any cells remaining in the supernatant and enumerated as described above. Viral and bacterial abundance, VBR, and the fraction of mitC-inducible lysogens (IF) were calculated as previously described (Williamson et al., 2005, 2007). In some cases, unreasonable estimates of burst size were obtained (e.g., BZ < 1) creating some doubt concerning the validity of the estimated IF. Therefore, IF was calculated with an assumed BZ and also expressed as the percent increase in viral abundance upon exposure to mitC relative to control samples.

Statistical analysis of the enumeration data was performed using the program Prism 6 (GraphPad Software, Inc., San Diego, CA). One-way ANOVA with repeated measures and a Tukey *post-hoc* test was used for pairwise comparisons to test the differences in bacterial and VLP counts across soils based on single extractions, and for differences in VLP abundance among multiple virus extractions.

### Terminal Restriction Fragment Length Polymorphism Analysis of Bacterial Community

As soil samples were stored at 4°C, total DNA was extracted from each soil sample using the PowerSoil DNA isolation kit (MoBio) according to the manufacturer’s instructions within 24 h after samples’ arrival. Terminal restriction fragment length polymorphism (T-RFLP) was performed as previously described (DeBruyn and Sayler, 2009). Briefly, triplicate polymerase chain reaction (PCR) was conducted for each sample to amplify 16S rRNA genes with 5 ng of extracted soil DNA as the template, 400 nM each of universal eubacterial primers 8F (tagged with FAM) and 907R (Lane et al., 1985; Reysenbach et al., 1994), and Premix Taq (Takara Bio Inc.). The following thermocycler program was followed: 95°C for 10 min, followed by 27 cycles of 95°C for 30 s, 45°C for 45 s, and 72°C for 1 min, and a final extension (72°C for 10 min). Purified PCR product (50–100 ng) was digested for 3 h with 10 U of AluI (Promega WI). Digested DNA was purified again then completely dried before 0.75 μl of ROX500 size standard (Applied Biosystems) and 15–30 μl deionized formamide were added to each sample (depending on concentration). After denaturation (96°C for 5 min), samples were analyzed on an ABI Prism 3100 Genetic Analyzer (Applied Biosystems) using default GeneScan parameters.

T-RFLP profiles of replicate PCRs were aligned by peak size, and peaks not occurring in all three replicates were removed. Total fluorescence for a trace was used to normalize replicates in a reiterative manner as previously described (Dunbar et al., 2001), and peaks less than 0.1% of the total trace fluorescence were removed. Consensus traces (average peak size and height) were then aligned and peaks within 0.5 nucleotides were binned. The resulting matrix was used for multivariate analysis and to calculate Shannon Diversity (H).

Canonical correspondence analysis was done using CANOCO for Windows version 4.54 (Plant Research International, Wageningen, The Netherlands, Lepš and Šmilauer, 2003). Predictor variables included soil edaphic parameters, IF, and VBR. Hill’s scaling with a focus on inter-sample distances and down-weighting of rare species was used, and a Monte Carlo permutation test evaluated the null hypothesis that ordination patterns were unrelated to environmental parameters.

### Randomly Amplified Polymorphic DNA Analysis for Viral Genetic Fingerprinting

Subsamples of virus extracts used for viral abundance were taken for RAPD-PCR reactions. Whole virus particles were used as templates for PCR reactions. Virus extracts (20 μl) were treated with 5 units of DNase I (RQ1 RNase-free DNase; Fisher Scientific, Pittsburg, PA, USA), 2 μl of 10× DNase buffer and incubated at 37°C for 30 min. This treatment was used to remove free DNA in viral extracts or concentrates (Allander et al., 2001). Reactions were terminated by addition of 2 μl of DNase I stop solution (Fisher Scientific, Pittsburg, PA, USA) and incubated at 65°C for 10 min.

The total volume of each RAPD-PCR assay was 25 μl, which contained 2.5 μl of 10 × Ex Taq Buffer (Mg^2+^ plus); 1.60 μl of dNTP mixture (2.5 μM each); 2 μl of 50 pmol primer; 0.50 μl of TaKaRa Ex Taq™ HS (5 units/μl; HotStart version; TaKaRa Bio Inc., Otsu, Shiga, Japan); and 1 μl of template (a supernatant containing 10^5^ virus particles). The primer used in this study was HCB-1 (5′-CCAGCAGCAG-3′) as described elsewhere (Srinivasiah et al., 2013). A single primer served as both forward and reverse primers in the PCR amplification. The thermal cycling conditions were as follows: (i) an initial denaturation at 94°C for 10 min, (ii) annealing for 3 min at 47°C, (iii) extension at 72°C for 1 min, and (iv) denaturation at 94°C for 30 s. Step (ii) through (iv) were repeated for 29 cycles; the run was ended with a final extension at 72°C for 10 min.

RAPD-PCR amplicons were separated on a 1.8% high-resolution agarose gel (Agarose – Hi-Res™ separation ≤1,000 bp; GenePure HiRes agarose; ISC BioExpress, Kaysville, UT, USA) made in 1X TBE (10X: 890 mM Tris base; 890 mM Boric acid; 20 mM EDTA; pH 8.3). Running buffer consisted of 0.5X TBE. Molecular weight markers were loaded into two terminal lanes and one central lane of the gel (200 bp DNA ladder plus; Fermentas Gene Ruler™, Burlington, ON, Canada). After electrophoresis the gel was stained with 1XSYBR gold (Molecular Probes Inc., Eugene, OR, USA) in 500 ml of 1X TE (10X: 100 mM Tris-Cl; 10 mM EDTA; pH 8) buffer for 30 min and de-stained in the same buffer without stain according to manufacturer’s protocol. Stained gels were imaged using the Kodak MI Imager (Amersham Biosciences, Buckinghamshire, UK).

Analyses of banding patterns were performed according to the method described previously (Srinivasiah et al., 2013). Briefly, gel images were analyzed using GelCompare II software (Ver. 5.0; Applied Maths, Sint-Martens-Latem, Belgium). Bands were initially selected by screening the fingerprints with 5% minimum profiling and 2% grey zone criteria before manually checking for band assignment. Matrices of banding pattern similarity were determined using Dice’s binary coefficient (Clegg et al., 2003). Dendrograms of banding pattern similarity were obtained using unweighted pair group method using arithmetic averages (UPGMA) (Williamson et al., 2008).

### Visualization Representation of Similarity Matrices From RAPD Cluster Analysis Using Multidimensional Scaling

The results of gel images using the GelCompareII software were compiled into two 30 × 30 similarity matrices of banding pattern similarity, one each for free and induced viruses. Each row and each column of these matrices represented a (month, site) pair, e.g., (May, T1) or (November, SF2). The (i,j)^th^ entry of each matrix x_ij_ represented the banding pattern similarity (expressed in percentage, with 100% denoting exact similarity) between the i^th^ and the j^th^ (month, site) pair, where i = 1, …, 30 and j = 1, …, 30. The objective was to obtain easily interpretable visual representations of the similarity data and use them to investigate whether free and induced viruses exhibited the same pattern of similarity/dissimilarity over months and sites.

Prior to performing the analysis, the *similarity* matrices were transformed to *dissimilarity* matrices by using the transformation y_ij_ = 100-x_ij_, so that the (i,j)^th^ entry in each transformed matrices now represented some measure of distance between the banding patterns for i^th^ and the j^th^ (month, site) pair. Obviously, both matrices were symmetric with zero diagonal entries. The multidimensional scaling (MDS) plot was obtained using the “isoMDS” function in library MASS of the statistical software R.

## Results

### Viral and Bacterial Abundance

**Figure 1:**
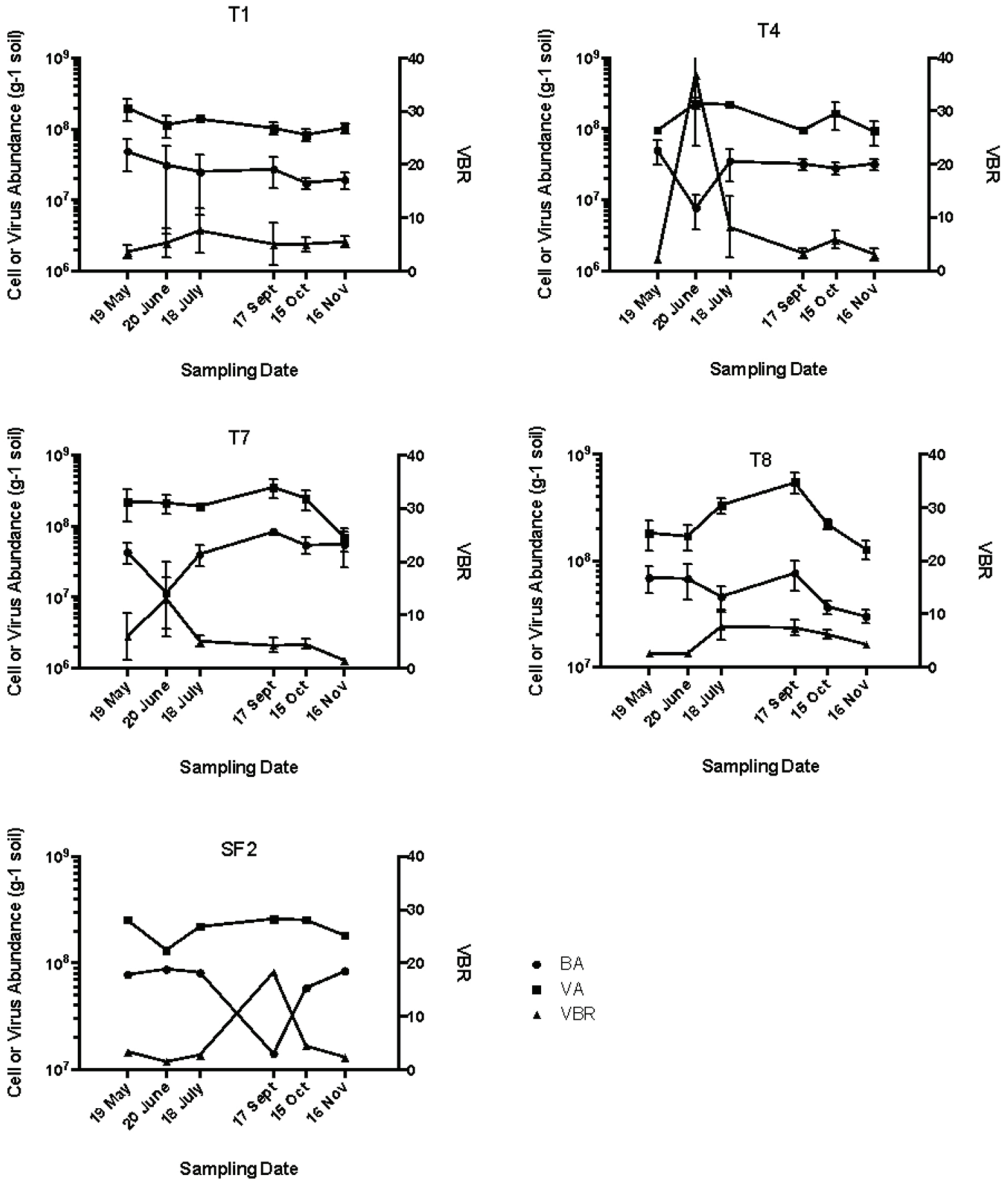
Bacterial abundance (BA), viral abundance (VA), and virus to bacteria ratio (VBR). T1, conventional till agriculture with corn-soybean-wheat rotation; T4, organically managed corn-soybean-wheat rotation with winter cover crops and no chemical inputs; SF2, forest; T7, early plant successional soil with annual burning; and T8, never tilled grassland with annual mowing.

In the agricultural soils T1 and T4, bacterial abundance was relatively stable from spring through fall at approximately 5.0 × 10^7^ cells g^−1^, except in the organically managed site (T4), where the bacterial abundance dropped to 7.0 × 10^6^ cells g^−1^ in June 2008 before returning to the previous level the following month (Figure 1). Viral abundance in both agricultural management systems remained relatively stable at approximately 1 × 10^8^ VLP g^−1^ soil from spring to fall 2008. The viral abundance typically exceeded bacterial abundance in both management practices by about 5–6-fold, except in June in the T4 soils where the VBR spiked to 40:1 before returning to a base level of 5:1 for all other sampling times. This increase in VBR was due to the decrease in bacterial abundance observed at this sampling site and time. The significance of the increase in VBR is unclear and may simply be due to an underestimate of bacterial abundance in these samples.

**Figure 2:**
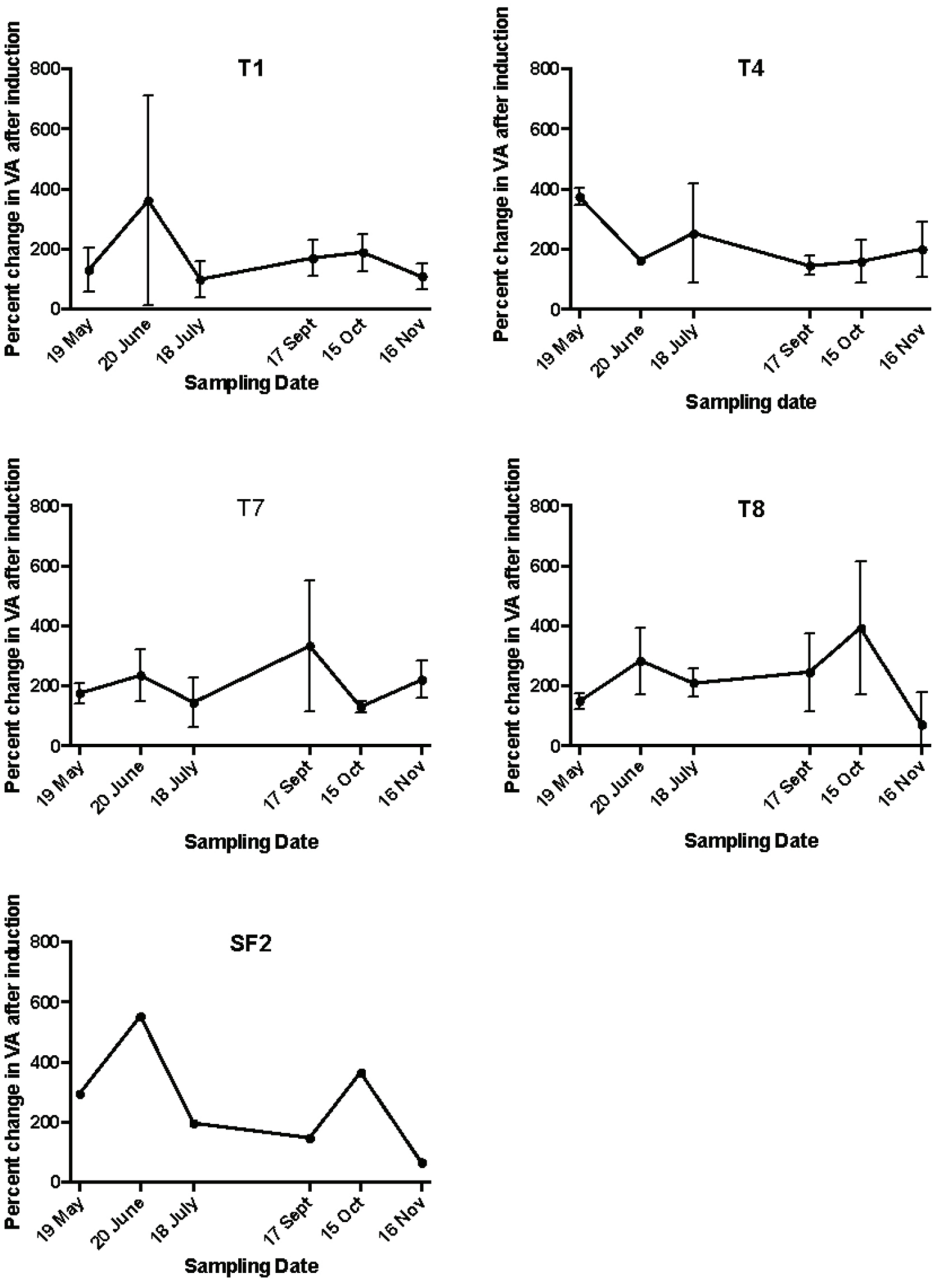
The percent increase in viral abundance (VA) in mitC-induced samples. The land management treatments are the same as described in Figure 1.

The mitC-IF is generally estimated by determining the increase in viral abundance and the bacteria lysed in mitC-treated and control samples, and then calculating IF by either assuming an average burst size or using the estimated measured burst size. In this study, the IF varied considerably depending on weather burst size was assumed or estimated in each sample (Table 1). The IF values varied seasonally and with land use when estimated burst sizes were used in the calculations. These values were deemed unreliable, however since the estimated burst sizes were quite low and, in some cases, estimated to be less than one virus produced per cell lysed. In these instances, cell growth of non-induced bacteria during the incubation period likely contributed to the low estimated burst sizes. When an average burst size of 20 was assumed and used in the calculation, the IF values ranged from a low of 0.9 to a high of 13% (Table 1). These values are considerably lower than those previously reported for soils and no discernable seasonal patterns were evident, although in the organically managed soil, T4, IF20 was highest in the spring (May) at 10.4%, decreased to 1.1% in July, and remained low throughout the remainder of the year. In the T1 soil, IF20 was highest in June at 13% and dropped to 1.3% in July and remained low throughout the remainder of the year. An alternative way to express induction results is by calculating the percent increase in viral abundance in induced samples relative to corresponding un-induced controls (Ghosh et al., 2008; Knowles et al., 2016). Though only a qualitative measure of IF, it does allow comparison of inducibility among the land use treatments and sampling dates. For T1 and T4 sites, positive induction events were observed on all sampling dates and ranged from 180 to 400% of control samples, with the highest rates of induction occurring in either May or June, but no discernible seasonal pattern was evident (Figure 2).

The seasonal distribution of bacterial and viral abundance in the early successional soils of T7 was similar to those observed in T4 with a spike in VBR in June due to low bacterial abundance values (Figure 1). The viral abundance values in T8 soil peaked in September, and the bacterial abundance values trended slightly downward from May to November. In the early plant successional soils T7 and T8, the VBR was consistently near 5:1 with the exception of the June sampling for T7 (Figure 1).

As in the agricultural soils, the early plant successional soils had low IF20 values ranging from a high of 2.35 and 4.4 in September for T7 and T8, respectively to lowest values of 0.89 and 0.41 for T7 and T8 respectively in November. No consistent seasonal trend was evident (Table 1). When expressed as a percentage increase in inducible viruses relative to non-induced control samples, positive induction events were observed for T7 and T8 sites at all sampling dates. The percent increase in inducible viruses ranged from about 50 to 400% but again, there was no observable seasonal pattern (Figure 2).

The absolute values of bacterial and viral abundance in the mid-successional forest soils were comparable to the agricultural and early successional soils except that the lowest bacterial abundance value in the forest was observed in September with a concomitant increase in VBR from about 4:1 to 40:1 during this sampling time. The IF20 values in the forest soil ranged from 0.62 to 3.14% (Table 1), and positive induction was observed at all sampling dates as measured by percentage increase in inducible viruses relative to un-induced controls (Figure 2). When measured in this way, two peaks in percent change in viral abundance after induction were observed in the forest soil SF2: the first in June with 600% increase and again in October with a 400% increase a pattern that differed from all other sites (Figure 2).

**Figure 3:**
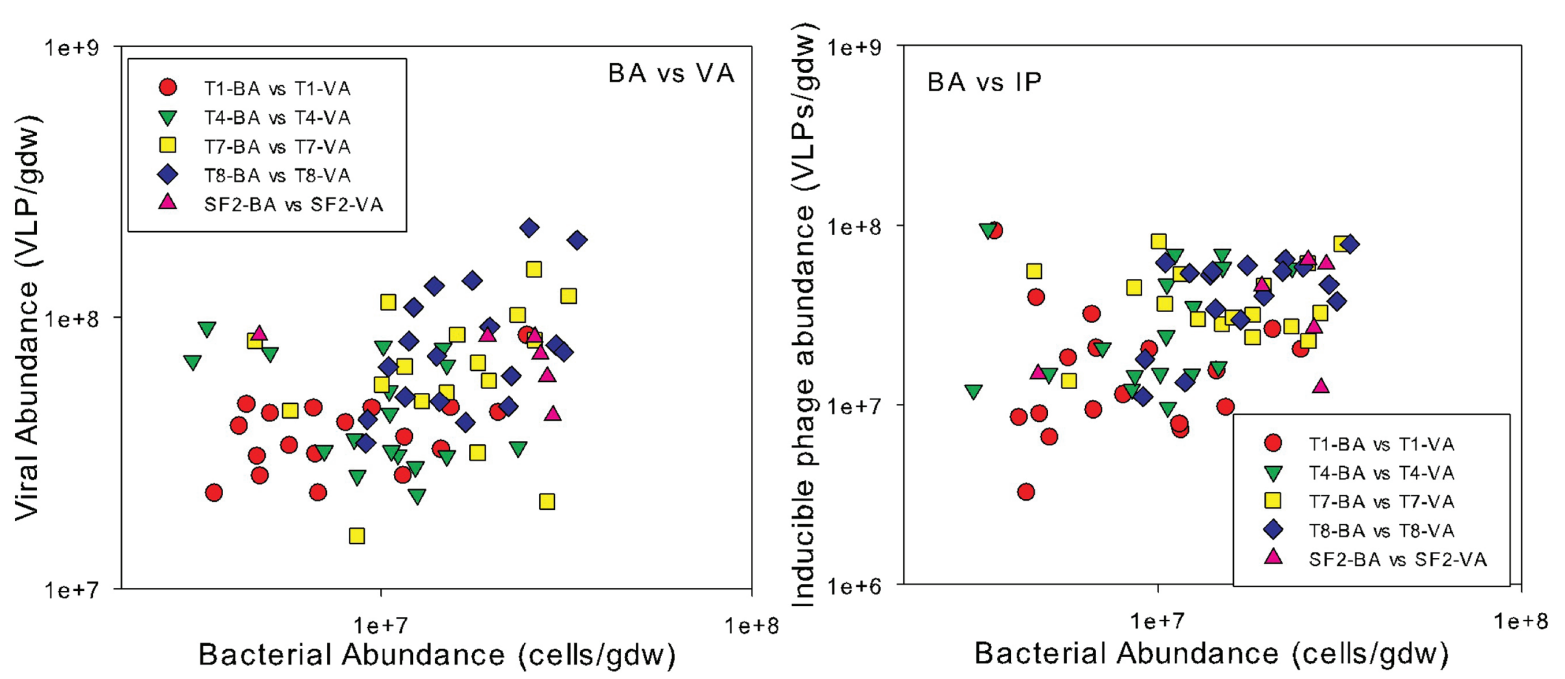
Relationship between bacterial abundance (BA) and viral abundance (VA, left) and inducible phage abundances (IP, right). The land management treatments are the same as described in Figure 1.

**Figure 4:**
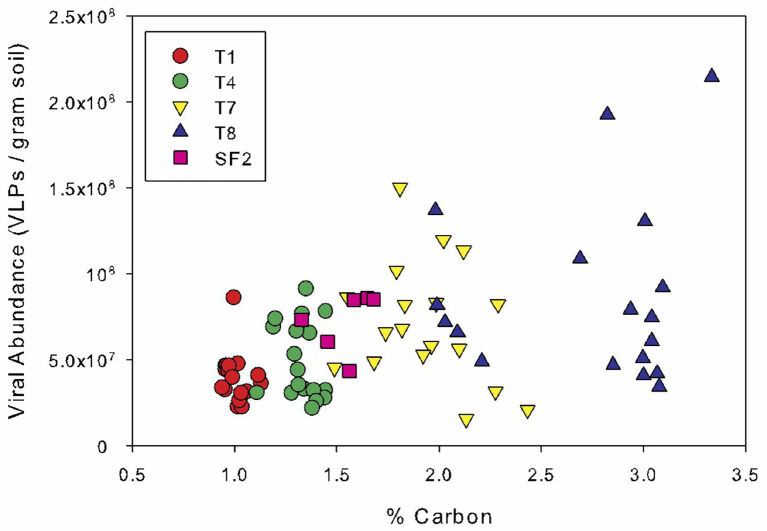
Viral abundance as a function of soil organic carbon. Data points are color-coded by land management type, and all dates are plotted. The land management treatments are the same as described in Figure 1.

Spearman’s rank correlation coefficients were calculated to reveal any significant relationships between the bacterial abundance, viral abundance, VBR, IF, and IF20 and soil edaphic properties. Across all sites and sampling times, bacterial and viral abundances were positively correlated (*r* = 0.331; *p* < 0.01) and both were also correlated to organic carbon content (bacterial abundance: *r* = 0.430; *p* < 0.001; viral abundance: *r* = 0.419; *p* < 0.001) and total nitrogen content (bacterial abundance: *r* = 0.373; *p* < 0.001; viral abundance: *r* = 0.386; *p* < 0.001) (Figure 3). Bacterial and viral abundances were also positively correlated to C:N (bacterial abundance: *r* = 0.439; *p* < 0.001; viral abundance: *r* = 0.362; *p* < 0.001). Interestingly, the seasonal variability in viral abundance at the five sites increased significantly with organic carbon content, as evidenced by the strong correlation between the standard deviation in viral abundance and organic carbon content (Figure 4). The IF was also correlated to moisture content (*r* = 0.234; *p* < 0.05). There was no correlation of the enumeration data with soil pH or phosphorus content. VBR was not correlated with any edaphic property measured.

### Terminal Restriction Fragment Length Polymorphism Analysis of Bacterial Diversity and Correlation With Other Soil Parameters

**Figure 5:**
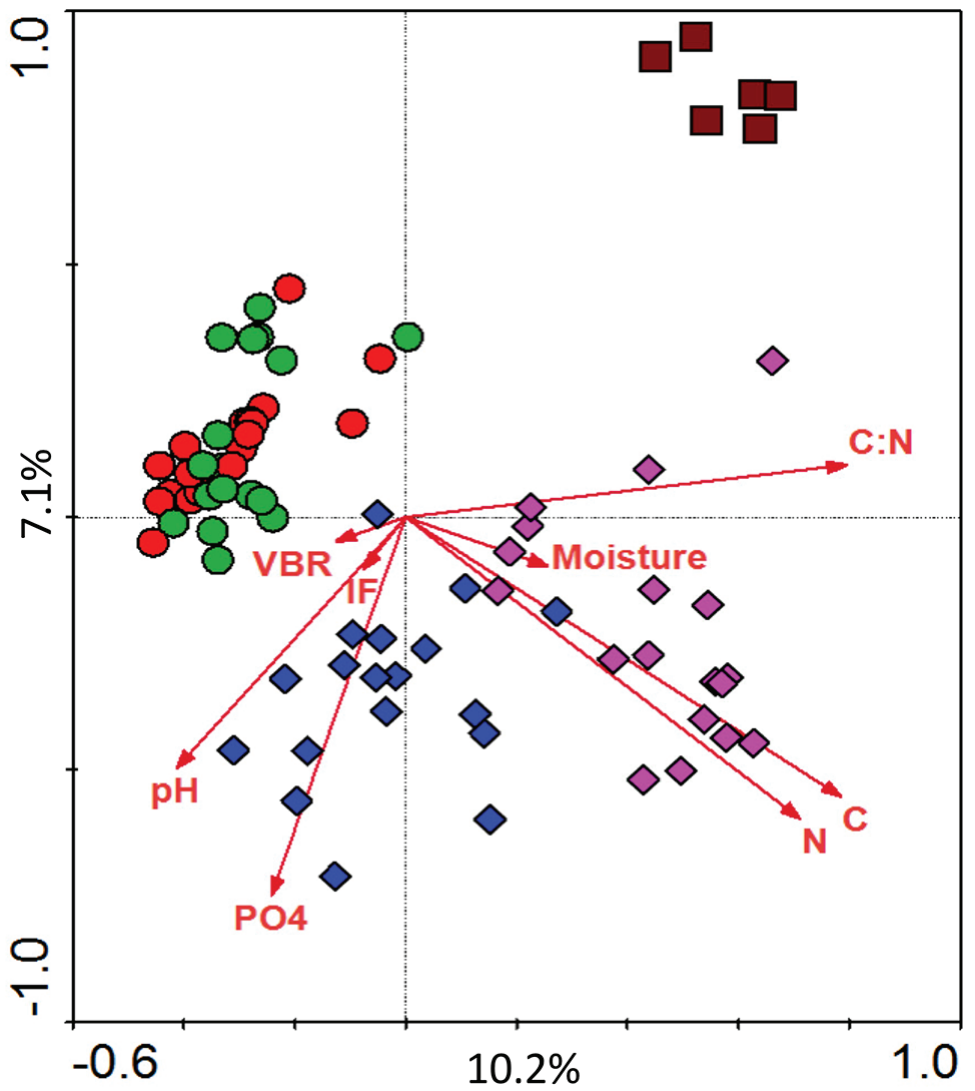
Diversity of bacterial community as determined using Terminal Restriction Fragment Length Polymorphisms (T-RFLPs). Ordination completed using canonical correspondence analysis (CCA), constrained by various environmental parameters (carbon, nitrogen, phosphate, moisture, C:N ratio). The treatments of soil samples were represented by colors and shapes. Red circle, T1; green circle, T4; blue square, T7; purple square, T8; dark red square, SF2.

For each plot replicate, triplicate T-RFLP profiles were generated and combined to yield a consensus profile. Technical replication between the triplicates was good (average Pearson *r* = 0.951). There was an average of 75 significant peaks per profile (≥0.1% of total trace fluorescence), which were binned into 168 unique TRFs across all samples. Average Shannon diversity (H) was 3.52 and ranged from 2.82 to 3.91; highest values were observed in the spring (May) and lowest in the fall (November). Canonical correspondence analysis of T-RFLP data constrained by soil properties (C, N, PO_4_, C:N, moisture, and pH) and community variables (IF and VBR) is shown in Figure 5. The primary correspondence analysis explains 10.2% of the variation, and the secondary explains 7.1%; Monte Carlo permutation revealed that the patterns are significantly different from random. The CCA revealed that land treatment was much more influential in structuring bacterial communities than sample date; or in other words microbial communities did not cluster by date (Figure 5). Instead, they appear to be structured by land management. For example, agricultural soils (T1 and T4) clustered together; in contrast there was separation by the other three land types (T7, T8, and SF2). This analysis also revealed greater seasonal variability in the early successional field plots (T7 and T8) compared to agricultural plots.

### Randomly Amplified Polymorphic DNA PCR Analysis of Viral Communities Across Different Land Management Sites

The RAPD-PCR genetic fingerprinting approach successfully generated reproducible genetic fingerprints that were strongly suggestive of spatial and seasonal difference in the composition of viral assemblages across sampling sites and dates. Unfortunately, the method was unable to deal with the plot-to-plot variability that existed within the land management treatments sampled over time. In other words, RAPD fingerprints from triplicate plots did not always cluster together. This limitation likely resulted from the inherent heterogeneity within our samples and the inability of our sampling design to adequately account for the heterogeneity rather than an inherent limitation of the RAPD-PCR approach to detect differences in the composition of the viral assemblages within our samples. However, to have a clear two-dimensional visual representation of the similarity patterns for free and induced viruses, MDS was used (Cox and Cox, 2008). The inducible prophage viral community structures had higher average dissimilarity (49.12) as compared to free extracellular viruses (34.03), although there appeared to be a single pair of free viral community structures that were extremely dissimilar (92.20). In other words, community structures for free viruses were, on average, more similar to each other than inducible prophage viruses.

**Figure 6:**
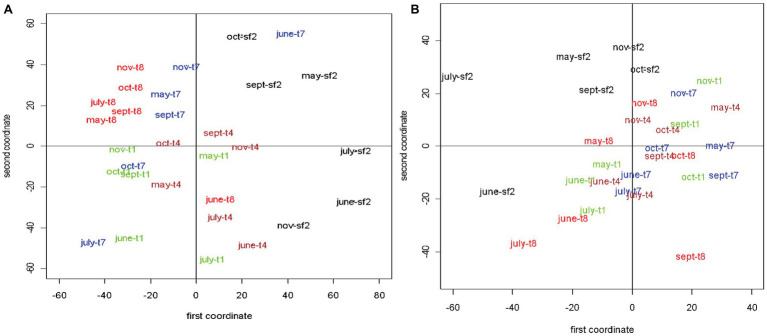
Multidimensional scaling (MDS) plots of the dissimilarity matrices based on the randomly amplified polymorphic DNA-polymerase chain reaction (RAPD-PCR) fingerprints for free extracellular **(A)** and mitomycin-C (mitC)-inducible viruses **(B)**. For ease of comparison, the five types of management sites are color-coded. The stress values associated with the two MDS are both less than 0.2. The land management treatments are the same as described in Figure 1.

Because the entries of the dissimilarity matrices represented distances among pairs of viral community structures, using the MDS technique, it was possible to represent the 30 data points [each corresponding to a (month, site) pair] as points in a two-dimensional space. Figures 6A,B show the two-dimensional visualizations of the dissimilarity matrices for free and induced viruses, respectively. For ease of comparison, the five types of sites have been represented by five different colors. Banding patterns obtained from site SF2 were different from the remaining four types of sites (Figure 6A) a pattern consistent with the separation of SF2 host bacterial communities from the agricultural and early plant successional sites. Except for the month of June, banding patterns obtained from sites T8 appeared to be most tightly clustered (least variable) over months from May to November. Also, the observations from T8 did not overlap with those from other site types except for the month of June. In addition, there seemed to be some overlap between observations from sites T1 and T4. Both of these types appeared to have less variability over time compared to T7. Observations from sites T7 were most dispersed and had some overlap with sites T1, T4, and even SF2.

## Discussion

Viruses are abundant and ubiquitous in soils from many environments. This study examined the temporal variability in virus-host population dynamics in agricultural, early plant successional, and mid-successional forest soils. In soils sampled approximately monthly undergoing five different land management practices, the viral abundance remained relatively constant from May to November at ca. 1 × 10^8^ viruses g^−1^ soil, a value comparable to previous studies. For example, the first direct enumeration of soil viruses using transmission electron microscopy estimated a total of approximately 1.5 × 10^7^ viruses g^−1^ soil in the sugar beet rhizosphere (Ashelford et al., 2003). Though this value was at least 350-fold greater than previous estimates using cultivation-dependent approaches with known host bacteria, controlled experiments with known titers of bacteriophage, suggested this TEM-based estimate may have still underestimated viral abundance by about 8-fold. A systematic evaluation of extraction conditions followed by direct enumeration using epi-fluorescence microscopy yielded counts ranging from 2.1 to 5.3 × 10^8^ viruses g^−1^ soil for agricultural soils in Delaware (Williamson et al., 2003) and in a later study examining a wider range of soil environments viral abundance ranged from 9 × 10^8^ to 4 × 10^9^ viruses (gdw^−1^) soil (Williamson et al., 2005). Viral abundance was correlated to soil moisture with wetland soil having the highest viral abundance. Interestingly, viral abundance and VBR were strongly correlated to differences in land use. Moist, organic matter rich forested soils showed high (>10^9^ viruses gdw^−1^) viral abundance. In contrast, dry and organic matter poor agricultural soils showed lower viral abundance (low 10^9^ to 10^8^ viruses gdw^−1^). Based on the limited studies available, temperate soil viral abundance appears to vary by about two orders of magnitude (~10^7^–10^9^) depending on soil type and environmental conditions (Williamson et al., 2017). However, unlike aquatic samples, viruses are difficult to recover from soils due to virus-mineral and virus-organic matter interaction that render the majority of extracellular viruses in a sorbed state (Kuzyakov and Mason-Jones, 2018). Thus, regardless of the extraction conditions, it is impossible to know the true extraction efficiency and so the recovered viruses likely only represent a sub-sample of the total virus population. The lack of standardized extraction procedure may account for some of the variability reported in the literature. Williamson et al. (2013) demonstrated that extraction conditions have a large impact on virus enumeration in soils. Nevertheless, for the extraction conditions used in this study, viral abundance appeared to be independent of seasonal variation, with the possible exception of viral abundance in the early plant successional treatment T8 (never tilled; annual burning) where viral abundance varied by nearly 1 log unit from May to November with the peak reached in September. Although successional patterns in the abundance of specific phages have been demonstrated (Ashelford et al., 2003), we are unaware of other seasonal monitoring studies of total viral abundance in temperate soils.

Generally, viral and bacterial abundance are tightly correlated in aquatic environments, with the ratio of viruses to bacteria remaining relatively constant at about 10:1 (Wiginton et al., 2016). In contrast, soil VBR values can be quite variable depending on the soil type and environment and can range from <1 to several thousand (Williamson et al., 2007; Parikka et al., 2017). The VBR values observed in the present study of ~5:1 were relatively low compared to other reported values and did not vary seasonally with a few notable exceptions (T4 and T7 in June; SF2 in September). Viral abundance is positively associated with the cooccurring bacterial abundance in the environment, since viral replication is dependent on bacterial growth and production (Liang and Radosevich, 2019; Narr et al., 2019; Liang et al., 2020). In treatments with viral abundance independent of seasonal variation, the bacterial abundance also remained similar regardless of seasonal changes. The relative constant bacterial production under seasonal variation may also be attributed to those months from May to November are all biologically active time periods for these soil systems. Virus-bacterial interactions are also related with viral replication strategies which may have important implications for viral and bacterial abundance.

In aquatic ecosystems we know that viral life-history strategies, lytic and lysogenic, vary with environmental stress and/or resource availability (e.g., trophic status) and have the potential to impact the quantity and quality of dissolved organic C in aquatic systems (Riemann and Middelboe, 2002; Breitbart et al., 2018). Lysogenic fractions vary widely (0.7–82%) between ecosystems, vary seasonally, and are associated with low production rates and low cell densities (Brum et al., 2016; Knowles et al., 2016). In a recent study in coastal Mediterranean lagoons, lytic reproduction was positively correlated with environmental conditions that favored strong physiological status of host cells, but lysogens were often undetected and highly variable (Maurice et al., 2013). Other marine surveys show that the lysogenic fraction is inversely proportional to host cell abundance (Brum et al., 2016; Howard-Varona et al., 2017; Liang and Radosevich, 2019), and that lysogeny is more prevalent in oligotrophic environments (Jiang and Paul, 1998) and in winter when productivity is reduced (Brum et al., 2016). In ice-covered lakes of Antarctica, for example, 89.5% of bacteria were lysogenic (Lisle and Priscu, 2004).

The seasonal variability of lysogeny in soil has never been studied. So the question still remains as to whether there may be a consistent seasonal succession in terms of viral reproductive strategies, and whether this succession might be driven by shifts in the abundance and physiological state of the bacterial hosts as in aquatic environments. In the present study, the IF, an index or estimate of the relative level of lysogeny in a microbial community, depended on how the IF was calculated. When using estimated burst sizes, IF values were generally higher than those reported for aquatic ecosystems and exhibited seasonal variation at some sites, with the highest levels observed in the fall and lower in the spring. However, in many instances, the calculated IF values were based on estimates of burst size that were unrealistically low, with sometimes less than one virus produced per bacterial cell lysed. When an assumed burst size of 20 was used to calculate IF, the values were much lower, typically 10% or less and did not exhibit seasonal variation with the lowest values observed in November. Based on these conflicting results, little can be concluded regarding the balance between lytic/lysogenic reproductive strategies and how they may vary with environmental conditions or host physiology and abundance in soil. However, based on the increase in viral abundance observed in samples exposed to mitC at all five sites and at every sampling time, it was evident lysogeny was detectable throughout the growing season from May to November, suggesting that lysogeny is a prevalent reproductive strategy for terrestrial bacteriophage.

The universal 16S rRNA gene has become the gold standard for assessing diversity patterns within microbial communities. Unfortunately, an analogous marker to analyze genetic diversity of viral assemblages currently is unavailable. As alternative approaches, PFGE and single marker genes conserved within specific groups of related viruses; the g23 gene of T4-like phages, for example, have been successfully applied (Filippini and Middelboe, 2007; Zhong et al., 2014) but suffer from distinct limitations. PFGE (i.e., electrophoretic separation of viral genomes) has limited resolution with considerable unresolved diversity within the limited number of bands on the gel (Jamindar et al., 2012) and specific marker gene polymorphism analysis can only target rather narrow specific sub-sets of viral communities. A genetic fingerprinting method with superior resolution and broad applicability to whole viral assemblages but without the expense and complex computational difficulty of high throughput sequencing is RAPD_PCR (Winget and Wommack, 2008). RAPD-PCR utilizes 10-mer oligonucleotide primers to randomly amplify viral DNA. Subsequent electrophoretic separation of amplicons of varying sizes generates rich genetic fingerprints of viral communities. Winget and Wommack (2008) used this technique to assess temporal and spatial variability of viral communities in Chesapeake Bay. In a temperate eutrophic lake, RAPD-PCR analysis revealed that viral communities were highly dynamic and exhibited strong seasonal shifts, and showed that viral and bacterial community composition were strongly linked (Hardbower et al., 2012). RAPD-PCR fingerprinting has also revealed that stormwater runoff can introduce terrestrial viruses to inland water bodies, driving changes in viral community composition during disturbance events (Williamson et al., 2014). In a previous study, RAPD-PCR was further improved by using viral metagenomes to inform selection of 10-mer primers and optimized for assessing genetic patterns in soil viral assemblages as a function of land use (Srinivasiah et al., 2013). In Antarctic soils sampled along a moisture transect, the composition of viral assemblages changed distinctly within a few meters. In the same temperate soils sampled for the current study, viral assemblages segregated by land-use (i.e., agricultural, plant successional, or forest soil) and all the KBS Michigan soils were more similar to each other than to an agricultural soil sampled in Delaware regardless of land use (Srinivasiah et al., 2013). To expand on the study by Srinivasiah et al. (2013), the RAPD-PCR genetic fingerprinting method was applied to assess seasonal variation in viral communities at the KBS land use sites. Potential differences between free extracellular viruses and mit-C inducible prophage were also examined. Though some additional optimization is needed to deal with the inherent plot scale heterogeneity of soil, the RAPD-PCR fingerprint analyses were suggestive of both spatial and seasonal variation in both free extracellular and inducible prophage viral assemblages. Most notably, viral assemblages of the SF2 forest site were clearly distinct from the agricultural and plant successional sites; a pattern that was also evident for bacterial communities as revealed by the t-RFLP analysis. This suggests that the composition of viral assemblages is linked to microbial host communities, a relationship that has been previously reported in aquatic ecosystems (Tijdens et al., 2008; Sandaa et al., 2009). Inducible prophage assemblages had a higher average dissimilarity than free extracellular viruses indicating a higher degree of seasonal variation. The difference was most acutely observed in communities of treatment T8. The greater variation in inducible prophage virus composition suggests that different sub-sets of the lysogenic microbial community were responding to mitC exposure at the various sampling dates. This assertion is supported by the large variability of the T8 microbial community based on the T-RFLP analysis. However, comparable seasonal variability in the microbial community structure was also observed in T7 soils but the opposite trend was observed for the free and induced viral assemblages. In other words, there was more seasonal variability in free virus than inducible prophage assemblages. The reason for this difference is unknown but may be related to different management practices employed to maintain the sites in early and mid-plant successional states for T7 and T8, respectively.

## Conclusion

Taken together the results of the study indicate that soil viral assemblages are highly dynamic and vary spatially and temporally, and are likely to impact the composition and function of prokaryotic host communities. Viral abundance is usually positively correlated with bacterial abundance, and soil organic carbon and nitrogen content can contribute to the variability in viral abundance. The structure of soil viral assemblages is influenced by land use and season, however free extracellular viruses and mitC inducible prophages may show dissimilar responses to the environmental factors. Moreover, there exist inherent difficulties in obtaining some important conclusions through field investigations. The high heterogeneity of soil physical properties, even within a single plot, makes it even more challenging for the field of environmental viral ecology to work at the field scale. Some questions about soil viruses may currently be better answered at the lab scale, where environmental factors can be more closely controlled.

## Data Availability Statement

The datasets generated for this study are available on request to the corresponding author.

## Author Contributions

KR, DG, and MR deceived the study. KR and DG performed the experiment. KR, DG, JD, TD, and KW analyzed the results. KR, DG, XL, and RW composed the article. All authors contributed to the article and approved the submitted version.

### Conflict of Interest

The authors declare that the research was conducted in the absence of any commercial or financial relationships that could be construed as a potential conflict of interest.

## Footnotes

^1^http://lter.kbs.msu.edu
